# Benefits and challenges of implementing statutory duty of candour in Victoria, Australia: a mixed methods analysis of healthcare provider perspectives

**DOI:** 10.3389/frhs.2025.1669958

**Published:** 2025-10-17

**Authors:** Reema Harrison, Corey Adams, Ramya Walsan, Elizabeth Manias, Ashfaq Chauhan, Nicole Youngs, Lanii Birks, Jennifer Morris, Liat Watson, Peter Hibbert, Ramesh Walpola, Jeffrey Braithwaite

**Affiliations:** ^1^ Australian Institute of Health Innovation, Macquarie University, Sydney, NSW, Australia; ^2^ Faculty of Nursing and Midwifery, Monash University, Melbourne, VIC, Australia; ^3^ Safer Care Victoria, Melbourne, VIC, Australia; ^4^ School of Health Sciences, University of New South Wales, Sydney, NSW, Australia

**Keywords:** statutory duty of candour, patient safety, adverse event, incident disclosure, apology, incident management

## Abstract

**Background:**

Statutory duty of candour (SDC) requires healthcare services by law to provide the patient or their family or carer who experiences a serious adverse patient safety event (SAPSE) with a written account of the facts, an apology, and the steps being taken to prevent reoccurrence. To date, the impact of SDC implementation has been understudied. As part of a state-wide evaluation of the impacts of SDC in the two years since its implementation in Victoria, Australia, this study focuses on staff and service delivery impacts of SDC.

**Methods:**

A mixed-methods design was employed, synthesising data from a 21-item survey with interview data. Health service staff working in Victorian health settings since SDC implementation in 2022 were recruited via state health agencies, professional organisations and social media. Survey data were subject to quantitative analysis using statistical software, with inductive content analysis applied to free text items. Reflexive thematic analysis was undertaken with the interview dataset.

**Results:**

A total of 170 respondents completed the survey, 25 of whom further participated in a follow-up interview. Survey participants were clinician managers (30%), nurses (24%), doctors (17%), allied health professionals (10%), and others (18%), primarily working in Victorian public (80%) and private (11%) hospitals. Staff reported greater awareness of SDC among staff with managerial responsibilities than frontline staff, with perceived gaps in staff knowledge about SDC and communication skills inhibiting practice. Seven themes further characterised the benefits, implementation challenges and implications of SDC: Promoting organisational accountability; Inconsistent event identification and review; Threshold for SDC is subject to interpretation; Prescriptive processes inhibit person-centred care; Context-specific implementation requirements; Adjusting to policy change; and Capacity and capability for implementation.

**Conclusion:**

Implementing SDC has contributed to greater structure, consistency and routine inclusion of patient and family perspectives when examining patient safety events. Opportunities for improvement identified by respondents and interviewees included developing person-centered and context-sensitive timeframes for communication, relaxing legalistic documentation requirements, findings ways to more consistently apply SAPSE definitions, and addressing the cultural implications and administrative burden of SDC requirements.

## 1 Background

Patients who experience harm because of the healthcare they are provided, and their families, often describe being deeply and detrimentally impacted (1). Physical, personal, financial, and psychological distress are frequently reported. But the lasting effects of poor communication and lack of openness by healthcare providers contribute to some of the most complex distress (2). Despite decades of research and advocacy, inadequate response to patient safety events remains a pervasive problem affecting the lives of patients, families and healthcare staff.

Recognition of the distress associated with inadequate communication and transparency about healthcare safety events ignited a range of policy and practice initiatives since the late 1990s worldwide. Extensive evidence has been generated during this time about the use of apology laws, along with disclosure and apology programs (3–5). In the US, many healthcare organisations adopted and evaluated disclosure and apology programs driven, in part, by a desire to reduce liability exposure and associated costs, whilst promoting learning from safety events (4). Contemporary approaches have shifted to Communication and Resolution Programs (CRP). Notably, CRPs extend beyond disclosure and apology, by including a focus on learning, peer support, and resolution or reconciliation (6). Such models have rarely been adopted beyond the US, with different models of health service delivery, clinical negligence contexts and health system financing in other settings shaping local implementation of approaches to facilitate communication and support around adverse safety events.

Over the last 20 years, a moral imperative for person-centred care in partnership with patients and families has underpinned policy and guidance about openness in the instance of healthcare harm. Resulting approaches have included the Being Open guidance released in the UK (7) and incorporating the Open Disclosure Framework (7) into Australia's National Safety and Quality Health Service Standards. Although a national requirement, numerous evaluative studies have highlighted the inconsistent application of the Open Disclosure Framework in practice (7–9). Such approaches to promote openness around adverse safety events reflect a global effort to promote information sharing and resolution following these events given the prolonged detrimental impacts on those involved (10).

Given the inconsistent application of open disclosure policy and guidance, patients and families have sought to shift policy and guidance into legislation in a bid to ensure they are told the truth about healthcare-associated harms. Consumer advocacy groups in the UK campaigned over several years for a Statutory Duty of Candour (SDC) that requires organisations and their executives to respond in an open and transparent way when things go wrong in care (11). SDC was legislated in UK NHS Trusts in 2014 and further NHS agencies in 2015, with similar approaches taken in other countries. Most recently, SDC has been implemented in the Australian state of Victoria in 2022 (12, 13). The threshold for candour varies by country from serious adverse safety events to all events in which there was potential for patient harm (11). Implications of non-compliance also vary between countries, with potential for criminal prosecution or further action from enforcement agencies in some settings (14).

In Victoria, SDC was introduced as part of a wider culture of change in health services across the state through the Victorian Health Legislation Amendment (Quality and Safety) Act 2022 (the Act) (12). SDC and protected reviews were recommended to foster an open and honest culture by elevating existing obligations of open disclosure and apologies. SDC applies in Victoria when moderate or severe harm occurs to patients. The Act introduced protections for adverse event reviews called serious adverse patient safety event (SAPSE) reviews, to encourage open and frank discussion between health services and consumers in such circumstances. Under the new legislation, if a patient experiences a SAPSE, the health service entity has a legal requirement to provide the patient or their family or carer with; a written account of the facts, an apology, a description of the health service's response to the event, and the steps being taken to prevent reoccurrence (12). As a relatively recent legislative shift, initial focus is on the actions taken to put SDC into place in the Victorian health system; its implementation, which is defined as “the act of starting to use a plan or system” (15).

Our recent systematic literature review reported that unlike incident disclosure initiatives, there is a lack of evidence about the implementation of a professional or statutory SDC specifically, and its impacts on patients, families, staff, and service delivery (16). A governmental duty of candour review is currently underway in the UK NHS (17), but no evaluative evidence has yet been published about the implementation or impacts of SDC. Now, over a decade after the first implementation of SDC internationally, there remains a significant gap in evidence about how SDC has been implemented, and whether the legislation has achieved the goals of promoting transparency, honesty and timely communication in responding to patient safety events.

Considering the evidence gap about impacts of SDC in care, and in response to a state-government requirement for evaluation, our transdisciplinary team of consumer, clinician, and academic researchers designed and conducted a comprehensive evaluation of the implementation of SDC in Victoria (18). Evidence of the perceptions, experiences and impacts of SDC on patients, families, staff and health service organisations were gathered in a series of studies. This article reports on a mixed-methods sub-study within this evaluation conducted with staff responsible for implementing SDC in Victoria. A further study will report the patient, carer and public perspective and experience of SDC implementation.

### 1.1 Aim

The aim of this research was to establish knowledge, experiences and impacts of SDC on the management of SAPSE in Victorian health services from the perspectives of healthcare staff as part of a broader evaluation. Specifically, we sought to achieve the following objectives:

1) To determine perceived knowledge, awareness and understanding of SDC among healthcare staff.
2) To establish the perceived effectiveness and impact of SDC in the management of patient safety events.
3) To identify the implementation challenges and barriers experienced by healthcare staff.

## 2 Methods

### 2.1 Design

Mixed method study synthesising survey and interview data from health service staff conducted as part of a broader evaluation described in detail elsewhere (18).

### 2.2 Sample

We sought to recruit 300 staff to complete surveys, with approximately 25 interviews to be conducted in follow-up. Target sample sizes sought to achieve a range of perspectives and experiences of SDC by geographic setting and service type in this exploratory work. A formal sample size calculation was not appropriate for this work. To promote anonymity and a voluntary approach, a self-selected sample was used. Self-selection occurred within the bounds of eligibility criteria that seek to limit the sample to those staff who had worked within relevant roles to SDC implementation in Victorian health services during the implementation period. Multiple channels were used in recruitment to identify healthcare staff with relevant roles in SDC implementation employed in a range of service types and locations in Victoria. Study information was shared via the Victorian agency with responsibility for healthcare quality; Safer Care Victoria (SCV), in addition to using social media, health professional networks, professional colleges as well as the research team's extensive network.

### 2.3 Data collection tools

A 15-item scale previously used to capture patient experience and outcomes of incident disclosure was used as the basis for the staff survey content (8). Items were reframed to reflect provider perspectives in relation to experiences of incident management in the two years since SDC implementation. Specifically, items were revised or added to capture the perceived impacts of SDC on practice. Additional items were added to align with the questions posed of staff in the UK SDC review to enable future comparisons to be drawn between experiences of staff in the UK and Australia. The final survey comprised 21 closed items and two free text items. Thirteen of the 21 closed items explored staff perceptions about knowledge, awareness and understanding of SDC among healthcare staff, leaders and consumers whilst the other 8 rated the perceived effectiveness and impacts of SDC in the management of patient safety events.

An interview schedule was developed informed by an interview schedule used in the UK Being Open policy evaluation (7) and applied with health service staff. Topics included awareness and understanding of incident management, personal experiences of SDC implementation, and its perceived effectiveness and impacts.

### 2.4 Procedure

To promote anonymity and confidentiality, the survey was administered via an anonymous online link using MQ Qualtrics software embedded in the study recruitment materials. Healthcare staff who wished to participate directly accessed and submitted the survey via this link. At the end of the survey, participants were provided the option to share their contact details to participate in an interview or to contact the project team directly to request an interview. Online interviews were conducted by a member of the project team (CA) experienced in conducting interviews about complex and sensitive health service topics. Microsoft Teams software enabled recording, transcription and secure storage of these data in the Macquarie University OneDrive for analysis.

### 2.5 Data analysis

Survey data were exported from Qualtrics into Stata 18 (Stata Corp, College Station, TX, USA) for analysis. Frequencies and percentages were calculated from the survey data using Pearson Chi-squared tests to explore between group differences by seniority, profession and health setting. A significance level of 0.05 was used for analyses.

Qualitative responses to free-text survey items about the benefits and challenges of implementing SDC were subjected to inductive qualitative content analysis (QCA; (19, 20) in Microsoft Excel. QCA, like other forms of qualitative analysis, focuses on understanding the meaning of phenomena and their contextual nuances, but also allows for some use of quantification, such as frequencies or percentages (2). Each response was read through and then classified using one or more brief descriptive codes in an adjacent column to summarise the content (1). As the classification process progressed, effort was made to re-use existing descriptive codes, where appropriate. After classifying all free-text responses, codes were read through to compare, refine and reduce them. From this a final set of codes, categories were developed and used to classify the content within all responses. These categories are reported with descriptions, illustrative quotes and frequencies among responses.

Qualitative interview data were managed using NVivo software version 12 (QSR International, Melbourne, Vic, Australia). Two researchers independently read the transcripts and conducted reflexive thematic analysis to inductively generate initial themes from key concepts. Initial themes were developed into a set of final themes through discussion with the wider team, including consumers, with consideration of our subjectivity (21). Themes were then labelled and reported in relation to the research aims. Through this process, we applied the principles of information power rather than seeking to achieve theoretical saturation as the latter was not considered appropriate to the diverse experiences, individuals and circumstances associated with adverse safety incidents (22). The full authorship team reviewed and agreed the final themes.

## 3 Results

### 3.1 Survey findings

Of the 212-healthcare staff who accessed the survey, 170 respondents completed one or more of the survey items (80% completion rate) and their data included in the analysis. Response rates for individual items ranged from 131 to 170. Table 1 shows the demographic characteristics of staff survey respondents, with 52% being frontline health professionals and 47% occupying quality management or patient liaison roles. Follow-up interviews lasting between 30 and 45 min in duration were completed with 25 of the survey respondents. Interviewees were clinicians who primarily occupied a variety of senior (*n* = 10, 40%) and middle (*n* = 8, 32%) management roles in quality and system safety departments with responsibilities for SDC implementation, along with clinical leaders in medicine (*n* = 3) and nursing (*n* = 1), in addition to doctors (*n* = 2) and nurses (*n* = 1) with interests in patient safety and experience of SDC implementation. Specific position titles were not reported due to the diverse roles with responsibility for SDC implementation and the risk of identification.

**Table 1:** Demographic characteristics of survey respondents.

Respondent characteristics	*n* (%)
Age group	
21–30 years	<5 (<2)
31–64 years	115 (67.6)
≥65 years	6 (3.5)
Unspecified	47 (27.6)
Gender	
Male	31 (18.2)
Female	85 (50.0)
Prefer not to answer	8 (4.7)
Unspecified	46 (27.1)
Professional role	
Doctor	29 (17.1)
Nurse/Midwife	42 (24.7)
Allied Health Professional	17 (10.0)
Healthcare manager (clinical manager, quality manager, patient liaison, risk manager)	80 (47.0)
Unspecified	<5 (<2)
Health service type	
Public Hospital	136 (80.0)
Private Hospital	18 (10.6)
Other services (ambulance/transport, community services, GP clinic, Outpatient settings)	10 (5.9)
Unspecified	6 (3.5)
Years in profession	
≤5 years	69 (40.6)
6–10 years	24 (14.1)
>10 years	71 (41.8)
Unspecified	6 (3.5)
Ever involved in a serious adverse patient safety event (SAPSE)	
Not involved/Unsure	26 (15.3)
Yes	136 (80.0)
Unspecified	8 (4.7)
SAPSE disclosed to patient/Carer	
No/Unsure	12 (7.1)
Yes	126 (74.1)
Unspecified	32 (18.8)

**Figure 1:**
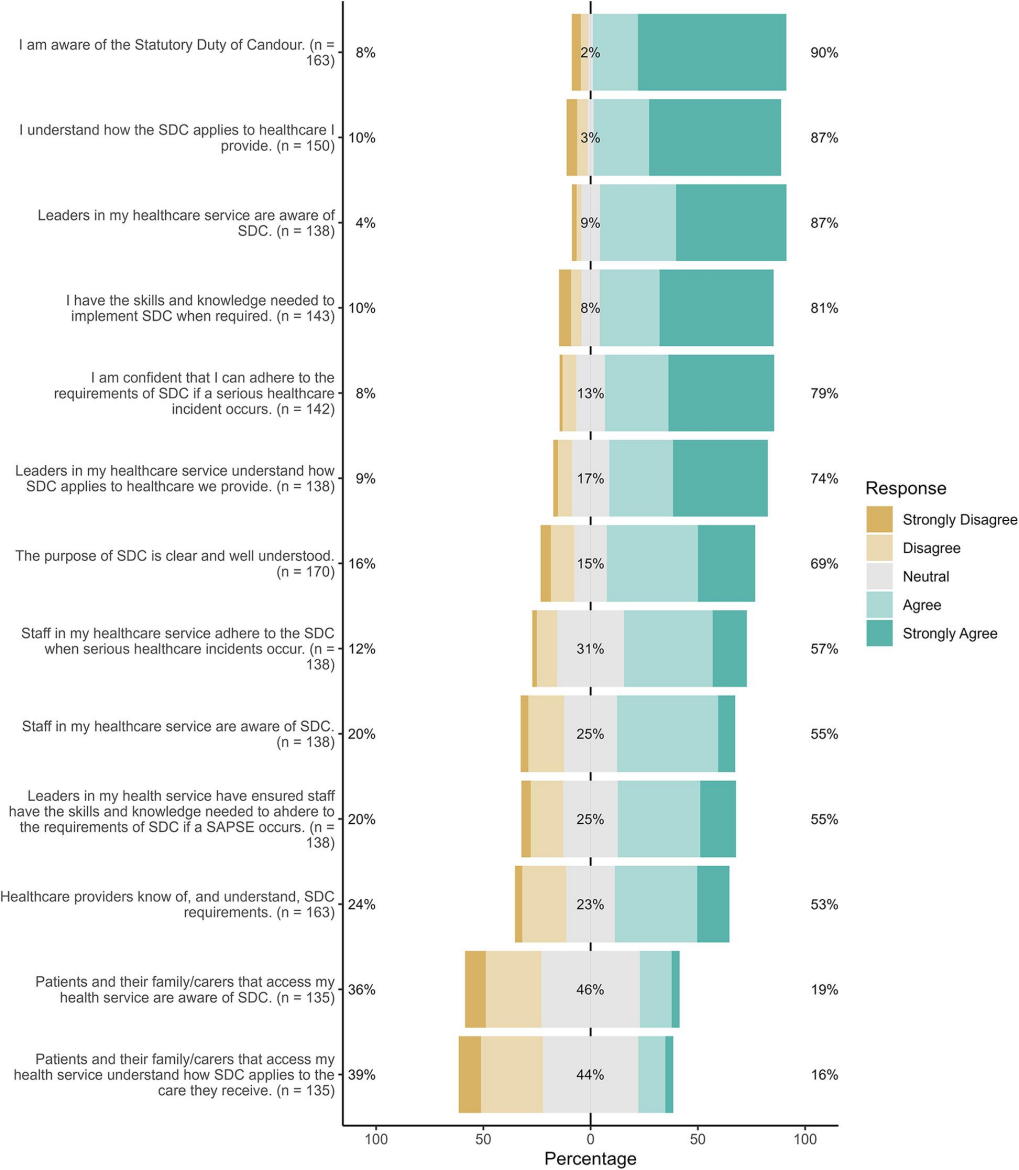
Perceived knowledge, awareness and understanding of SDC.

Figure 1 provides the full Likert distribution of responses to 13 of the 21 items that explored staff perceptions about knowledge, awareness and understanding of SDC among healthcare staff, leaders and consumers. Each bar is segmented to show the proportion of respondents who hselected Disagree or Strongly Disagree (left-hand portion), Neutral (middle section), and Agree or Strongly Agree (right-hand portion). The number of respondents (*n*) for each item is displayed beside its label. Overall, most respondents (81%; *n* = 138) indicated that the purpose of SDC is well-understood. While a majority (87%; *n* = 112) agreed that leaders were aware of SDC, only 55% (*n* = 76) reported that leaders have ensured staff have adequate skills and knowledge of SDC. Notably, nearly a quarter (24%, *n* = 39) of respondents indicated that healthcare providers do not know of or understand SDC requirements. Just over one-third (36%, *n* = 49) of respondents believed their patients were not aware of SDC. Similarly, 39% (*n* = 53) indicated that patients do not understand how SDC applies in their care. Significant differences in perceptions were identified between those in different professional roles, healthcare settings and genders. Those in managerial roles, working in hospital settings and/or were females reported significantly higher levels of agreement on all items (*p* < .05).

**Figure 2:**
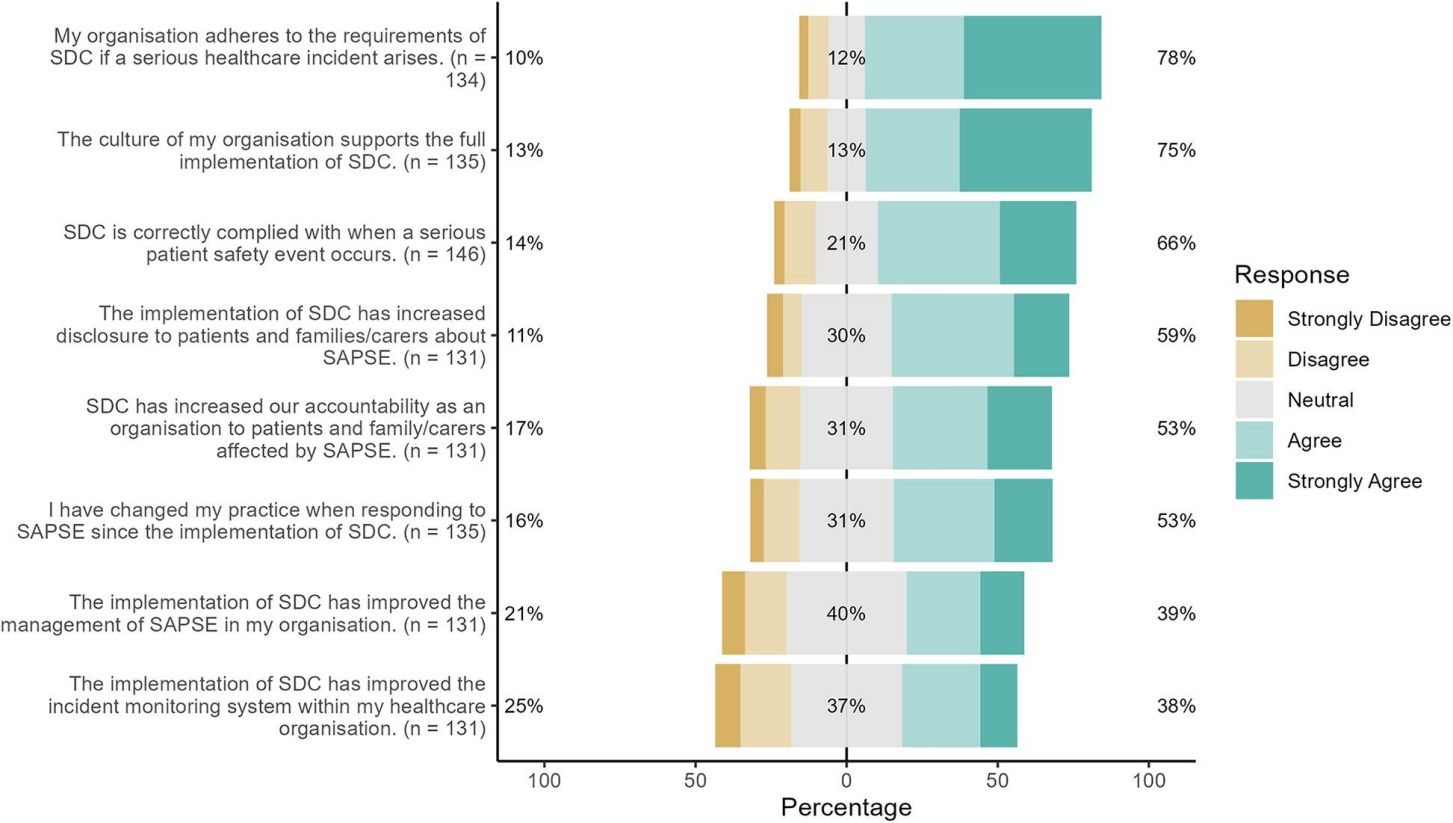
Perceived practice changes from SDC implementation.

Figure 2 depicts findings from 8 of the 21 survey items about the extent to which practice change was perceived to result from SDC implementation. Many respondents (78%; *n* = 114) believed that their organisation adheres to the requirements of SDC if a SAPSE arises, that the culture of their organisation supports the full implementation of SDC (75%; *n* = 101). However, a smaller proportion (66%, *n* = 96) reported that SDC is correctly complied with in practice. Significant differences in responses to these items were identified between those from different professional roles, settings, and by gender. Staff working in private hospital settings, who identify as female or occupy a managerial role were more likely to agree with these statements (*p* < 0.05).

Just over half 53% of respondents (*n* = 71) indicated that their own practice had changed when responding to a SAPSE. Similarly, 53% (*n* = 69) indicated that organisational accountability to patients and families had increased since the implementation of SDC. Fewer (39%, *n* = 51) agreed that SDC had improved the management of incidents within their organisation or (38%, *n* = 50) incident reporting. Significance differences were again noted by professional role, setting and gender such that those in managerial roles, public hospitals or identified as women reported greater agreement with the statements (*p* < 0.05).

Free-text survey comments further elaborated on both perceived benefits of SDC in safety event management and the implementation challenges. This was an important additional data source to capture qualitative data about SDC implementation from staff who wished to remain completely anonymous and did not participate in an interview. Table 2 provides a synthesis of findings of the perceived improvements in practice associated with SDC that were reported by survey respondents. The numerator is the number of respondents that made comments under the given theme, and the denominator is the number of survey respondents that provided free text comments. Survey respondents predominantly identified greater procedural structure, consistency, and promoted consumer involvement in incident reviews as benefits resulting from SDC implementation. Table 3 synthesises qualitative responses from free-text items in the survey about the implementation challenges associated with SDC. Tensions between rigid timeframes, documentation requirements, and person-centric care were commonly reported as barriers to implementation. Several practical barriers to implementation were also identified relating to the administration of SDC exemplified in Table 3.

**Table 2:** Free-text categories of perceived benefits of implementing SDC.

Category	Description	Extract	*N* (%)
1. Building trust with families, with transparency and honest communication.	The SDC process could help to facilitate communication and transparency, which could help to rebuild trust with between clinicians and patients and families following an adverse event.	“Open honest communication to the patient about their care” “Transparency and honesty is the best policy” “Trust and transparency” “The opportunity to be given an apology for what they have experienced gives the family information to move on as required.” “More transparency… better understanding/closure for families”	*35%* (*36/102)*
2. Increased family involvement in the SAPSE review process.	A key benefit was increased involvement of patients and families in the review process. This provides families with the opportunity to share their perspectives about the incident, share the emotion impacts, and ask clinicians specific questions about the adverse event and management. Clinicians also highlighted that SDC process could provide additional support to families during this challenging time.	“Involvement of patients families and carers and opportunity to express their thoughts and ideas following an adverse event.” “Providing the patient/family with a structured means of having input into the review” “This includes a greater opportunity for patients and their families to share patient impact statements or submit questions to be answered during the investigation process” “Providing a family with an opportunity to ask questions about a serious adverse event” “It allows for some humanity and acknowledgement of the emotional experience of harms.”	*20%* (*21/102)*
3. Increased organizational accountability for management of SAPSEs.	Clinicians perceived SDC as a method of increasing organizational accountability in response to serious adverse events, which is strengthened by the legislative backing of SDC.	“Greater accountability and transparency between health services and their consumers.” “More accountability to fully investigate serious incidents” “To ensure healthcare providers are accountable for any incidents that occur.” “Legislative backing to implement the process with patients and families”	*15%* (*16/102)*
4. Improved consistency and structure of SAPSE review process	One of the benefits of SDC was creating a more standardized process for SAPSE review. Although some clinicians reported that they had good existing processes for reviewing adverse incidents, they recognised the benefits for other organizations that may have lacked rigorous processes and/or structures.	“I understand, unfortunately, that this legislation is necessary however because this practice doesn't always happen and the legislation provides additional structure and recourse around the need for open disclosure.” “Consistency in how incidents are managed” “We were already doing this. But I understand that some were not.” “Standard and vigorous approach to managing SAPSEs and open disclosure.”	*9%* (*9/102)*

**Table 3:** Free text survey data of implementation challenges.

Category	Description	Extract	*N* (%)
1. Rigid timeframes may be unsuitable and lack patient-centredness.	Clinicians consistently raised concerns about the mandated timeframes associated with SDC, particularly the requirement for early contact with family members. While well-intentioned, this timing was often perceived as misaligned with the emotional and contextual needs of families, especially those experiencing grief. As a result, several clinicians reported that this requirement to initiate contact within a fixed period could cause distress or be perceived as insensitive. Clinicians suggested the need for more flexibility for timeframes, particularly for initial conversation, which could be guided by the individual needs and readiness of the patients and families.	“Timelines are not person-centered. ” “The timelines are unachievable and often not considerate in times when a patient has passed away. We are trying to contact families whilst they are grieving and arranging funerals” “Inflexible timeframes—patients/family members may not be ready…or need some time to prepare psychologically and take things at their own pace.” “The time limits imposed. In many circumstances, it's too soon for patients or their families to think and engage.”	*34%* (*38/110)*
2. Formal opt-out creates unnecessary burden for families.	The opt-out process was seen as being an unnecessary legal requirement, and not patient-centric. Alternative approaches were proposed, such as documenting phone conversations, rather than completing a form.	“Mandatory to opt out in writing—feels too legalistic and defensive” “Meeting the timeframes, when consumers/patients do not want to participate but do not sign the opt out form” “Having to provide written consent to not take part is not patient centred” “We have found the decline form is very legal, and families that decline SDC also decline signing the form… A less legalese letter would help”	*5%* (*6/110)*
3. Negative impact of SDC Process on families.	Clinicians also highlighted that some families may experience the SDC process negatively, which may be a lengthy and emotional process, with unclear resolution for families.	“I think it sometimes makes the patient/family's experience worse in honesty.” “The rigidity of the process, the formal language of the written responses—these can seem cold and un-human to patients… it can drag out and be detrimental to their experience I think” “Traumatizing consumers or NOK when reviews don't find root causes or end up being the trajectory of illness. It's a traumatic experience putting someone through the process”	*3%* (*4/110)*
4. Inconsistent implementation of SDC, including thresholds for SAPSE.	Clinicians reported some confusion and inconsistency about the thresholds for defining a SAPSE. In particular, clinicians highlighted confusion about thresholds for surgical complications (that were discussed during the consent process). There may also be pressure to not define an incident as “serious” (SAPSE) to avoid having to undertake SDC review. Some felt like there were no clear guidelines, and there may be inconsistent implementation across different healthcare organizations.	“No clear guidelines. Some organisations blur the guidelines around SDC so only open disclosure is completed not full SDC. If we don't call it a SAPSE we de don't have to do SDC” “There are different interpretations of what requires SDC. The examples from SCV provide some guidance, but situations are unique. My impression is that different health services have adopted different approaches…” “The significant grey areas of what is a SAPSE and what is not.” “Reporting timelines and clarity regarding what is an APSE vs. a SAPSE particularly in relation to incidents within the residential aged care setting and some returns to theatre.” “Understanding the indication to begin the process, particularly when a complication from a recognized risk occurs and had been discussed in detail during work up for a procedure and during the consent process”	*8%* (*9/110)*
5. Cultural misalignment with SDC processes, including blame and fear of punishment.	Clinicians reported fear of being blamed, or punitive actions resulting from SDC. There may be considerations about potential litigation or reputation risks to the organization. There were several comments about leadership/management lacking admission for medical errors, and lack of consultation with clinical staff. Rather than feeling protected by SDC, there is an impression that staff may be more vulnerable to blame or punishment if admitting to errors.	“A challenge is trying to change learned behaviours and a sense of fault and blame, an overarching “I don't want to be sued” legal lens.” “Culture. Some leaders still can't admit to an error. Where the leadership is open and encouraging and supportive, the staff follow” “Hierarchy more interested in covering up or diluting information of incidents to protect theirs and organizational reputation” “It can be a punitive/blame game rather than a learning experience that improves patient care” “Ego, worry about protecting self or colleagues” “Staff do not want to admit fault because they might get into trouble”	*10%* (*11/110)*
6. Lack of staff awareness and training about SDC.	Clinicians often reported the need for more staff awareness and training. Whilst general awareness building seemed to be supported, as part of open disclosure training. However, there were some mixed opinions about varying staff training needs, with specific training for people who are involved in SDC process—i.e., training required for more senior staff, not all clinicians.	“Education is required for all clinicians at all levels” “All staff understanding SDC and having the conversational intelligence/skill to communicate with patients/families/carers” “For healthcare staff to gain in depth knowledge and understanding of the process and ability to support or communicate this to patients at the point of care. SAPSE's may not happen regularly so staff aren't routinely exposed to the process.” “It's a hard thing to do and people need training and support to implement” “I don't believe the whole organization(clinicians) need to know how to do SDC because their focus should be on the open disclosure. The SDC follows and is focused on the review—that is not a clinical function.”	*8%* (*9/110)*
7. Increased administrative burden due to SDC processes, with lack of necessary resourcing.	Increased demands from SDC, particularly around administrative burdens for documentation, and coordination of activities such as SDC meetings. Clinicians reported a lack of resourcing to support these additional tasks. Resourcing has not expanded in alignment with job demands, including multiple demands for rural clinicians.	“Administrative burden (implementation and ongoing) is not sufficiently resourced.” “No change in funding or resourcing from the Department for the additional workload required” “Completing reviews within the specified timeframes. This is primarily related to resourcing” “Coordinating meetings and meeting the timelines” “Strict timeframes to adhere to that can be unrealistic for our organisation with limited staff and resources to complete these, especially in regional and rural health services…”	*8%* (*9/110)*
8. Psychological impacts of staff involvement in SDC meetings.	Clinicians also highlighted the psychological impact of SDC on staff members, particularly negative implications from meetings with family members and feelings blamed for adverse events. This was described as being very stressful and having implications for staff mental health, with lack of support for staff who are involved in SDC.	“The unintended negative effects of the process on the mental health of the staff taking part in the SDC process” “Managing family's emotions, blaming staff (managers) who have to sit there and apologise which is incredibly difficult … Allowing the SDC meeting for families to abuse or yell at staff, and they just have to sit there and take it…Not having appropriate supports post SDC to support staff. As this is also very difficult for the affected staff involved as managers.” “Stressful meetings added to already stressful job” “The unintended negative effects of the process on the mental health of the staff taking part in the SDC process”	*3%* (*4/110)*

### 3.2 Interview findings

Eight themes were generated from the interview data that enrich the survey findings. Resulting themes were: (1) Inconsistent event identification, (2) Threshold for SDC is subject to interpretation; (3) Services lack capacity to administer SDC; (4) Clinical workforce requires skillset to administer SDC, (5) Promoting organisational accountability; (6) Prescriptive processes inhibit person-centred care; (7) Creating space for patient and family perspectives, and (8) Context-specific implementation requirements.

#### 3.2.1 Inconsistent event identification

This theme described the challenge posed to healthcare providers in identifying all SDC eligible events through the multi-source information available. Respondents described three main information sources for event identification: (1) organisational incident reporting systems, (2) complaints management systems, and (3) from patients experiencing problems with their care. Interviewees described a variety of methods for identifying eligible events from these sources and ensuring an appropriate response. Methods ranged from checking only the most severely coded events in organisational reporting systems, to all events reported via these systems, and expanded into a wider review of multi-source data including patient-led identification. The latter was preferred by several experienced quality managers despite being time-consuming. Challenges were identified in dual identification of SAPSE and sentinel events. Ultimately, the same event occurring in different services may not be identified to be considered for SDC.

“ISR ones and two are obviously always flagged for discussion, so it's generally at that meeting where we would make that final decision.” (HP15)

“We look at all our ISR ones and twos, so they would be SAPSE.” (HP11)

“Now we actively look for SAPSE in our M and M (Mortality and Morbidity) meetings, in our complaints.” (HP2)

“This whole SAPSE on top of Sentinel events has created utter confusion” (HP9)

“We do not automatically classify an ISR one or two as a SAPSE” (HP26)

“Often … our SAPSE are then also confirmed and reported as Sentinel events to Safer Care Victoria, which means then there's new timeframes for the sentinel reporting, which can be quite challenging” (HP10)

“We look at every single incident that comes through the doors, and sometimes that can be up to about 1,500 to 1,800 a month… to identify incidents that might meet the threshold for being a serious incident that has caused significant harm to a consumer, and it was likely preventable” (HP20)

#### 3.2.2 Threshold for SDC is subject to interpretation

The threshold for implementing SDC with identified events was inconsistent between healthcare providers. For some, the degree of patient harm (in Victoria, Incident Severity Rating or ISR) was used. Events that were coded as more severe because they led to patient harm or had potential for serious harm were all assigned to SDC. For others, events that met the threshold for SDC were instances in which the organisation was at fault; for example, when an incorrect process or practice occurred. When focusing on fault, this constrained the implementation of SDC in the context of complications of care. Overall, due to the thresholds for SDC, it was apparent that the same event occurring in different services may not trigger SDC or protected reviews.

“We have started a SAPSE review group to clearly identify those that may or may not fall … with a more formal approach.” (HP1)

“It's tricky [to decide if SDC applies] when you've obtained informed consent for a procedure and then they happened to experience one of the complications” (HP26)

“If it's a return to theatre that had poor escalation, it automatically would fit into a SAPSE and if it was a return to theatre with appropriate and timely escalation of care, then it it's not so” (HP1)

“If you've got a legal person in the room, everything's a SAPSE.” (HP9)

“So, anything that falls as incident severity rating one or two through our incident management system, we would review and ascertain as to whether it falls under a SAPSE or an APSE^1^.” (HP1)

“Our ISR ones are all classified as protected SAPSE.” (HP11)

“The definition [for SDC] was very grey at the start. I think as we've gone along and matured as an organisation, we have greater understanding of moderate harm, and it depends on the actual care the patient's receiving” (HP13)

“You read through this when you're debating whether an event is a SAPSE and it can be 50/50 in a room full of people” (HP14)

#### 3.2.3 Services lack capacity to administer SDC

Organisational capacity for SDC implementation was raised by most respondents, along with staff capability to conduct the process effectively. Staff from some organisations described having SDC leads or dedicated staff within quality management teams, whilst others described SDC responsibility as within the scope of work for consumer relations staff. Regardless of where SDC responsibility was positioned, respondents converged that significant resources were required to administer the legislation, presenting challenges for their overstretched clinical workforce.

“Resource constraints, staffing, shortfalls are a massive contributing factor…it's across the organisation and whether it's attrition due to illness, increased workloads, they all have an ability to impact” (HP10)

“It causes a lot of work because in our private sector, the Director of Clinical Services is sort of like the general manager, the patient safety manager, the quality manager and also sometimes a little bit of the operations manager and the finance manager and that's [what its'] like [in] these small facilities, it's huge, a huge role” (HP3)

“I am the duty of candour coordinator … my role has evolved a lot over the last couple of years… it was more just ensuring that…we are meeting the legislation in terms of KPIs…my role now is very much more case management.” (HP15)

Events that were eligible for SDC were more common than sentinel events but described as requiring similar resources. Beyond identifying eligible events, resources were required to organise meetings, contact experts, communicate with patients and families, and develop reports.

“The coordination of stakeholder engagement has also proven to be challenging in terms of the reviews…. we have a panel who does the review and nominating panel chairs and external subject matter experts having a consumer on the panel…then with any report…we involve our legal counsel”(HP10)

Several staff commented that administering SDC may be counterproductive due to the resources consumed, with these challenges exacerbated in rural areas. Concerns were raised by some staff about the considerable time spent on SDC work in organisations that applied SDC for known complications of care.

“The staff that I'm asking to do this work can be part of these review panels have farms and stock” (HP1)

“[SDC] it's taken my time and other people's time to meet these key criteria away from things that we could actually be prevented” (HP1)

“If you identify a SAPSE, that's just a complication of healthcare that was unexpected, but unpreventable… it puts in process a whole lot of irrelevant work with sometimes thousands and thousands of dollars worth of staff hours looking into something which provides no benefit… It wastes a lot of time reviewing how this happened when it was just a known complication that happens” (HP20)

#### 3.2.4 Clinical workforce requires skillset to administer SDC

Beyond organisational capacity to deliver the SDC process, limited staff capability in communication, review and report writing skills were identified as barriers to effective SDC implementation. Through describing their diverse methods, respondents reported lack of clarity about the most appropriate review method to select, using diverse methods including the London Protocol and Root Cause Analysis (RCA). The increased frequency of reviews being conducted within services since SDC implementation posed challenges for identifying suitably skilled staff to engage in review processes.

“We've got lots of people that can do in-depth case reviews, but it actually does require expertise to go through that process to be able to really unpack what happened, pinpoint the critical events and investigate them really critically… I think if when you don't have that expertise, the investigation can be too superficial” (HP26)

Gaps in the communication skills required of the clinical workforce to engage in SDC communications were identified. Nuanced knowledge of where, when and how to conduct SDC communications was described as essential but lacking. Several respondents suggested that a SAPSE or SDC coordinator would be beneficial, along with coaching for staff engaging in SDC to administer the process effectively.

“So I think there is something about having someone more senior (in the meeting) who's got more confidence and expertise to be confident enough to say I'm sorry this happened” (HP26)

“But you must be mindful that not everyone's a good communicator. Surgeons are notorious for being not good communicators at all… So I think we need just basic training, human factors training, or just common language training that everyone has to undergo.” (HP19)

#### 3.2.5 Promoting organisational accountability

Despite variations in the implementation of SDC between providers, respondents consistently discussed that SDC legislation had fundamentally changed the management of safety events. At an organisational level, the requirement for SDC was described by many as elevating the visibility of safety events to senior clinician leaders and executives. Respondents suggested that this visibility was a step towards more consistent organisational accountability for such events.

“I think it's (SDC) given structure for accountability because I don't know if that was necessarily there before” (HP3)

“We have a group meeting with our Chief Medical Officer and clinicians and senior clinicians. We look at the IR ones and twos, sometimes we need more information” (HP2)

Experiences differed between services and health settings, but the need to adhere to timelines within the legislative requirements was a central driver for many respondents. Although timeframes for SDC activities were often discussed as a challenge, they were also identified as valuable for holding the organisation to account.

“When it's legislative. Everyone is sort of worried about the timelines and adhering to the timelines and what if it doesn't meet the timelines” (HP11)

“It brings a sense of urgency with strict timelines that you have to maintain… when I reported to the Chief Medical Officer, anything that overshot the timelines was investigated as to why are we not meeting deadlines. So I think it holds people to account” (HP12)

#### 3.2.6 Prescriptive processes inhibit person-centred care

Many described a tension between adhering to rigid timeframes and their ability to be responsive to patient and family needs. Most interviewees converged on the perception that the stringent and inflexible SDC timelines were barriers to person-centric and appropriate communication. Three specific communication requirements were frequently identified as barriers to person-centric care. The first was the need to communicate about an event with patients and families within 24 h, which was not considered appropriate in some circumstances. Examples provided included an event identified post-discharge or during a holiday period.

“The prescriptive timelines for when you tell patients about an event, a SAPSE can be highly inappropriate, but there is no allowance in the legislation or the guidelines for flexibility.” (HP20)

“I've seen patients become, or families in particular, become very anxious and paranoid during this process because the information comes at a time when they're at a heightened state of distress.” (HP20)

“Those timelines, which pressure us to contact the patient with the family within a certain timeframe, we're forever having to debate should we give it a go or should we deliberately breach those guidelines.” (HP16)

“But the whole 24 h, it’s not enough time for people to gather enough facts to have a sensible conversation. And I think patients find it a bit confusing and confronting in 24 h” (HP23)

Secondly, the short timeframe for a review and report to be completed was identified as difficult to meet because of challenges in identifying events that require SDC, the extensive work required for a comprehensive review, the limited availability of staff with suitable review and report writing skillsets. These challenges were heightened in regional or rural settings in which access to suitably qualified staff and those involved in the event were further constrained.

“It's the contact with the consumer within three days, and it's the report within 50 days. Oh, look, I just can't highlight that enough” (HP10)

“Once a SAPSE has been identified, which might be immediate or it could actually be retrospectively months down the track, depending on the scenario… the problem that comes from those identifications is that it obviously leads to the processes required by duty of candour” (HP14)

The third major barrier presented by stringent SDC processes was in the requirement for patients and families to complete opt-out paperwork to be excluded from the SDC communication requirements. Interviewees described instances in which families were discontent with the care process and did not wish to be engaged by the service as presenting a tension between person-centred care and meeting statutory requirements. By enforcing opt-out paperwork, including physical signatures from these families, staff were unable to meet family wishes to not receive further contact.

“The legislative requirement for written opt out and the suggested statement from the department on how to opt out sounds very legalistic.” (HP16)

“If you don't want to have anything to do with the process, you're also the least likely to want to fill in another form about the process you don't want to do.” (HP5)

“To be honest, probably 90% of them [families] have declined being involved in SDC” (HP22)

#### 3.2.7 Creating space for patient and family perspectives

In some services, the introduction of SDC meant a substantial departure from existing practice. Requirements for SDC have created space for patients and families to be routinely informed about the findings of a review and provide their perspectives. Igniting discussion with consumers about their care was identified as a positive change in practice by many, leading to nuanced understanding of events, their causes and impacts.

“Generally our in-depth reviews didn't involve the consumer or their family. It was probably more of a look at the records and talking to the staff who are involved. Whereas the SDC process requires us to at least offer that to the consumer or their family.” (HP15)

“Yeah, we had an in-depth case review process, but we did not have a process whereby we incorporated all the feedback from consumers into that review process. And that's a key change.” (HP11)

“I think probably our reviews weren't as robust because you get so much more nuanced information around exactly what happened by hearing it direct from the patient or their family member that you're never going to get from staff or from looking at the notes.” (HP23)

“Before, it was a bit uncomfortable sometimes, so it was easier to maybe sometimes not involve the consumer. But now we have to and therefore we go through that process.” (HP15)

In other services, SDC was seen to formalise existing good practice and scaffold this activity through the requirements of SDC legislation. Specifically, these respondents described greater consistency in their communication structure with consumers due to the stringent SDC communication requirements.

“It almost depends a lot on what the preexisting of in quality culture was rather than SDC really creating a change in that culture” (HP9)

“We've got a summary report that we give, and it's a page and a half, and it's written in consumer-friendly language” (HP10)

#### 3.2.8 Context-specific implementation requirements

Misalignment was reported between the SDC process and care pathways for patients who receive care beyond an inpatient setting at a single metropolitan site. Respondents from regional and rural contexts highlighted the challenges for SDC implementation within the current requirements in geographically distributed communities. Key challenges were the difficulty in identifying and assembling suitably qualified staff to conduct reviews, particularly during periods of natural disaster, such as bush fires or flood, and during holiday periods. The high volume of regional and rural patients accessing care distributed between major centres and local services was a notable challenge, especially when events were identified post-discharge.

“So the timelines are not realistic for a regional setting… The implementation of the statutory duty of Candour has just provided another level of complexity when we're already stretched as a health service” (HP1)

“We (rural facilities) are always bound by the same legislation… I understand that when we are making a legislation change or a quality change, it needs to be considered across the state… Regional centres are probably the biggest risk because we don't have access to platforms or to staff to do things” (HP1)

“But then you know, we have bushfires. Last week, everybody had to cancel their meetings. So you know it, it doesn't it it's not easy to get those panel meetings” (HP20)

Contextual challenges to SDC implementation were also identified in relation to the delivery of private health care due to the visiting medical officer (VMO) model and nature of the relationship between the service and medical care providers. In private hospitals, leaders described their need to engage VMOs and encourage them to deliver care in their services because they are not employed staff. The power dynamic and employment relationship between VMOs consulting in private services was therefore influential in their ability to involve this group of staff in SDC processes. Incidents that occurred within private hospitals or multi-site delivery between public and private hospitals were therefore identified as challenging to navigate by respondents who were quality and safety leads.

“When we're doing statutory duty of candour and doing open disclosure, we can't force a doctor to go and do open disclosure… It can be really challenging when we've got our accredited practitioners who are not obliged to do open disclosure, and then we are trying to meet these requirements so we can do our part of open disclosure” (HP3)

## 4 Discussion

Whilst SDC has been endorsed and encouraged in health systems over a decade ago, evaluative evidence of the implementation or impacts of SDC on patients, families, staff and healthcare delivery has been lacking (16). Our mixed methods evaluation has sought to contribute essential evidence to address this gap (18). In gathering survey and interview data from staff involved in SDC implementation in Victoria, Australia about their experiences in the two years since its introduction, this study provides novel insight into the perceived gains and implementation challenges experienced.

In taking a highly structured approach underpinned by legislation, respondents with responsibility for quality and safety management suggest that SDC has provided greater consistency in the way in which patients and families are communicated with following a SAPSE. In their services, requirements for information sharing, apology and provision of written information were described as having influenced the degree to which patients and families are engaged by health services about safety events. Yet a myriad of implementation challenges was identified that appear to constrain staff in the implementation of SDC and its ability to meet the desired outcomes of the legislation. Whilst many respondents reported their organisation adhered to the requirements of SDC in response to SAPSE, inconsistency in the selection of events defined as SAPSE and the decision to engage in SDC was highlighted. Some services appear to base their decisions on apparent fault or perceived liability as opposed to degree of harm, which defines the threshold in Victorian guidelines. The types of implementation challenges encountered are reflective of those identified in the context of SDC in the UK (16), and broader communication and resolution programs in the US (23, 24).

Differences in the perceptions of respondents about their knowledge and awareness of SDC, along with the perceived changes in practice that have resulted through its implementation, were noted by professional role, setting and gender. Those in managerial roles and public hospitals reported greater agreement with the survey items, perhaps reflecting their greater exposure to adverse safety events in these positions and settings. Whilst safety events may arise in private hospital contexts, complications of care are often addressed in the public hospital system in Australia. The reason for different experiences reported by genders is unclear and does not appear to be associated with the job roles occupied.

Implementing SDC in Victoria seeks to achieve several goals, namely, promoting transparency, openness, honesty about safety events with affected patients and families (12). At present, the available incident management data do not enable the examination of quantitative data post-SDC implementation to the extent of implementation or whether these goals have been achieved. Our findings, through self-reported data—suggest that where SAPSE are appropriately identified, SDC appears to be influencing organisations positively in terms of making affected patients and families aware of the event, providing written information about what happened, and providing an apology. SDC enactment is however contingent on the appropriate identification of events. Despite widespread acknowledgement of the limitation of incident reporting systems (25), our analysis (and wider work) indicates healthcare organisations remain heavily reliant on retrospective incident reporting to identify SAPSE (26). Whilst system-wide indicators of successful SDC implementation would be valuable for evaluating its uptake and impacts, there are no current mechanisms in place to provide these data available or planned.

Event definition and detection are pervasive challenges (25). In our study, respondents indicated that patient complaints, patient reporting and medical record review mechanisms were also being used to identify SAPSE, reflecting wider literature (27, 28). Although a broader range of incidents may be captured by these mechanisms, staff in our study identified significant resourcing challenges. Methods to promote incident identification to enable SDC, including patient-identified events, remain needed. Appropriately resourced and sustainable review approaches are required to enable adequate response to the high volume of events that require communication with patients that meets the intend of the SDC legislation (29). Opportunities to leverage artificial intelligence (AI) to integrate and assess complex unstructured data may warrant exploration are a mechanism to support identification of events from multiple data sources (30).

Many respondents suggested that SDC implementation had not shifted their practice in the management of SAPSE and that the legislation simply provides greater structure. Yet both nationally and internationally, disclosure of safety events to patients and families remains inconsistent and of variable quality (31). Respondents in the study appeared to focus on the procedural steps in response to a SAPSE rather than whether cultural change in openness about incidents and genuine engagement with patients and families. Given the ongoing reluctance of healthcare providers to identify adverse safety events and mistakes in care, these perspectives warrant further scrutiny (32). Our findings identify the need to review flexibility, timeframes, processes for identifying and reviewing events, and opt out mechanisms in initial steps to advance SDC implementation, with further work required to ensure appropriate event identification, patient and family engagement.

### 4.1 Limitations

Conducted as part of a broader analysis of the implementation of SDC in Victorian health services, study findings must be considered in context and with reference to the limitations of the research. Data were gathered from healthcare professionals who volunteered to respond to the survey and, for some, to also participate in a follow up interview. Survey responses were received from a range of health professionals, with interviewees from managerial roles overrepresented in the qualitative sample. Interviewees were primarily those with responsibility for implementing SDC to some extent in their services. These managers provide a valuable perspective of the implementation process and challenges relevant to the study aims, but their perspectives about the success of the implementation must be considered in context of the wider evidence generated in this work and from others about the experiences and perspectives of other affected stakeholders, namely patients, families and frontline workers. The limitations of a self-selected sample apply here and reflect an ongoing challenge within this field of highly sensitive research. Self-selection was necessary for an anonymous and voluntary survey. Given the topic, the option for complete anonymity and to not participate in an interview was however appropriate. In conjunction with this study, a further manuscript is in development that reports the patient and family experience of SDC implementation in Victoria. This research was conducted in one Australian state, and findings are relevant to SDC implementation in the context of Victorian policy and practice. Relevance to further health settings may be explored in further comparative analyses. Whilst our survey tool was adapted from a survey previously used in the context of open disclosure, no formal validation methods were applied.

## 5 Conclusion

For many Victorian health services, the introduction of SDC has intensified their existing Open Disclosure mechanisms by providing greater consistency and structure to organisational processes. Greater structure has been accompanied by challenges of rigidity and lack of person-centredness. Despite the potential gains associated with SDC, limited and inconsistent event identification, coupled with challenges in implementing review processes, present hurdles to ensuring that the right patients and families receive the information, apologies and explanations they deserve when met with a healthcare safety event.

## Data Availability

The raw data supporting the conclusions of this article will be made available by the authors upon reasonable request, and subject to appropriate ethical approval to access these data.

## Footnotes

^1^Adverse Patient Safety event
